# Adding Three Cycles of CAPOX after Neoadjuvant Chemoradiotherapy Increases the Rates of Complete Response for Locally Advanced Rectal Cancer

**DOI:** 10.3390/curroncol28010033

**Published:** 2021-01-06

**Authors:** Zhiwei Zhai, Kunning Zhang, Chen Wang, Tian Zhang, Lixia Wang, Jiannan Yao, Zhenjun Wang

**Affiliations:** 1 Department of General Surgery, Beijing Chaoyang Hospital, Capital Medical University, No.8 Gongren Tiyuchang Nanlu, Chaoyang District, Beijing 100020, China; zhiweizhai@ccmu.edu.cn (Z.Z.); wctj2018@163.com (C.W.); 2 Department of Pathology, Beijing Chaoyang Hospital, Capital Medical University, Beijing 100020, China; kunkunzhu@msn.cn; 3 Department of Radiotherapy, Beijing Chaoyang Hospital, Capital Medical University, Beijing 100020, China; ztscience@163.com; 4 Department of Radiology, Beijing Chaoyang Hospital, Capital Medical University, Beijing 100020, China; wlxchaoyang@163.com; 5 Department of Oncology, Beijing Chaoyang Hospital, Capital Medical University, Beijing 100020, China; yjnchaoyang@163.com

**Keywords:** rectal cancer, total neoadjuvant chemoradiotherapy, consolidation chemotherapy, pathological complete response

## Abstract

Background and Objectives: the total neoadjuvant chemoradiotherapy (TNT) includes different strategies, but the most appropriate model remains uncertain. The purpose of this retrospectively study was to evaluate the safety and pathological response in the consolidation chemotherapy model. Methods: patients with cT3/T4 or TxN + M0 rectal cancer that were receiving neoadjuvant chemoradiotherapy (CRT) (50 Gy with oral capecitabine)/TNT (CRT followed by three cycles of CAPOX) during September 2017 to September 2019 in our department were included. All of the patients were recommended to receive radical surgery. Results: a total of 197 patients were included. Eighty-one patients received CRT, while one hundred and sixteen patients received TNT. Nine patients did not undergo surgery because of the distant metastases (one patient (1.2%) in CRT group, two patients (1.7%) in TNT group) or a refusal of resection (two patients in CRT group, four patients in TNT group). The pathological complete response (pCR) rate was 32.7% in TNT compared with 12.8% in CRT (*p* = 0.002). There was no statistically significant difference in grade 3 acute toxicities of neoadjuvant treatment and surgical complications between the two groups. Conclusions: the consolidation chemotherapy model is safe for patients with locally advanced rectal cancer and it has a high pCR rate. The long-term follow-up is necessary to be evaluated in a future prospective, randomized trial.

## 1. Background

The standard therapeutic approach for locally advanced rectal cancer (LARC) (T3/T4N0, or TanyN1/N2) is neoadjuvant concurrent chemoradiotherapy (CRT), followed by total mesorectal excision (TME). Postoperative adjuvant chemotherapy is also recommended in these patients. This multidisciplinary treatment has significant improvements in reducing the risk of local recurrence [1,2]. However, distant metastasis now exceeds the rate of local failure, which is the primary cause of cancer death in rectal cancer [3]. Adjuvant chemotherapy for rectal cancer does not show a clear benefit in improving the overall survival (OS), disease-free survival (DFS), or distant recurrences [4,5,6]. One meta-analysis of over 6000 patients suggested that patients with a pathologic complete response after chemoradiotherapy may not benefit from adjuvant chemotherapy, whereas patients with residual tumor showed superior outcomes when adjuvant chemotherapy was given [6]. In addition, the patient’s ability to tolerate adjuvant chemotherapy was poor [7,8]. Nearly 30% of eligible patients had never started adjuvant chemotherapy [9] and less than half of them had received the full chemotherapy or initiated treatment after a significant delay [7,10]. Therefore, there is controversy regarding the efficacy of adjuvant chemotherapy in LARC.

Several researchers have reported the total neoadjuvant chemoradiotherapy (TNT), which means to move the adjuvant chemotherapy to the preoperative setting [11,12,13,14,15,16]. It has the promise to better address microscopic metastatic disease early and increase treatment compliance. The current study defined TNT as induction chemotherapy, followed by CRT or consolidation chemotherapy (delivering systemic chemotherapy after CRT). However, which is the most reasonable TNT model is still unknown. There were many studies on induction chemotherapy, but few on consolidation chemotherapy [17]. It has been previously reported that, in patients undergoing CRT with consolidation chemotherapy, tumors are less likely to regain metabolic activity between six and 12 weeks [18].

The aim of this retrospective study was to evaluate the safety and pathological response in the consolidation chemotherapy model.

## 2. Materials and Methods

### 2.1. Patients

We included 197 patients with LARC who underwent CRT or TNT during September 2017 to September 2019 based on hospital coding at the Department of General Surgery, Beijing Chao-Yang Hospital. Patients that were aged at least 18 years, with diagnosis of rectal adenocarcinoma by biopsy and clinical stage Ⅱ (T3-4, N0) or Ⅲ (any T, N1-2) who had a distal tumor border within 12 cm from the anal verge by rigid proctoscope were eligible for inclusion. Clinical staging was done by endorectal ultrasonography, pelvic magnetic resonance imaging (MRI), and computed tomography (CT). The eligible patients were required to have an Eastern Collaborative Oncology Group performance status score of 0 or 1and normal bone marrow /liver/kidney function. Patients were excluded if they had the following characteristics: (1) presence of distant metastases; (2) presence of unresectable cancer; (3) previous chemotherapy or pelvic radiation; or, (4) previous history (within five years) of malignant tumor.

Informed consent was obtained from all of the participants before treatment. The study was approved by the ethics committee of Beijing Chao-Yang Hospital, Capital Medical University (Number: 2016-350). All of the procedures that were performed in studies involving human participants were in accordance with the ethical standards of the institutional and/or national research committee and with the 1964 Helsinki Declaration and its later amendments or comparable ethical standards.

### 2.2. Procedures

All of the patients received pelvic intensity-modulated radiotherapy (IMRT) with concurrent oral capecitabine at 825 mg/m^2^ twice daily. Patients in the TNT group received three cycles of consolidation CAPOX after chemoradiation. Subsequently, a radical surgery was undertaken at least three weeks after the completion of consolidation chemotherapy. The mean interval was 12 ± 1.2 weeks in TNT group. Patients in CRT group had surgery 6–8 weeks rest after chemoradiation. Patients underwent IMRT receiving 2.0 Gy/fraction per day, five days per week, for a total dose of 50.0 Gy in 25 fractions. Consolidation CAPOX consisted of oxaliplatin 130 mg/m^2^ on day 1, capecitabine 1000 mg/m^2^ twice a day on day 1–14, every three weeks.

Both of the groups all underwent repeat coloscopy, endorectal ultrasonography, CT, and pelvic MRI restaging at one week before surgery. The tumor response was assessed by Response Evaluation Criteria in Solid Tumors (RECIST) guidelines in solid tumors [19] before surgery. Patients who developed distant metastasis during the neoadjuvant treatment course received systemic therapy according to the decision of the Multiple Disciplinary Team (MDT). Patients with clinical and radiological evidence of complete clinical response (cCR) were still recommended to receive radical surgery. A wait-and-see approach was only conducted in patients who refused radical surgery. Surgery was done according to the principles of TME. The surgical procedure, including abdominoperineal resection (APR), low anterior resection (LAR), or Hartmann procedure (Hartmann) was planned ahead appending on the height of the tumor. However, intraoperative complications could change the course of action.

### 2.3. Pathologic Examination

The 7th edition of the American Joint Committee on Cancer (AJCC) TNM system was used for staging [20]. After TME, ypTNM stages were evaluated by two experienced pathologists, where it was a standard practice in our hospital. The system used to grade tumor response was recommended by the AJCC cancer Staging Manual modified from Ryan R [21]. The pathological complete response (pCR) was defined, as there were no residual cancer cells in the resection specimen [22].

### 2.4. Outcomes

Patient characteristics, neoadjuvant administration and toxicities, pathologic response, operative approaches, results, and complications were retrospectively analyzed. Adverse events during total neoadjuvant setting were measured while using the Common Terminology Criteria for Adverse Events version 5.0 (CTCAE5.0). Postoperative complications were defined as any medical or surgical complication that occurred within 30 postoperative days.

### 2.5. Statistical Analysis

The statistical analysis was performed with the Statistical Package for the Social Sciences software, version 20 (SPSS Inc, Chicago, IL, USA). The continuous variables are expressed as mean ± standard deviation (SD). Student’s t test, Chi-square test, and Wilcoxon test were used for statistical analysis. *p* values < 0.05 were considered to be statistically significant.

## 3. Results

### 3.1. Patients Characteristics

Of these 197 patients, 81 patients were treated with CRT and 116 patients were treated with TNT. Three patients did not undergo surgery because of the distant metastases (one patient (1.2%) in CRT group, two patients (1.7%) in TNT group). Six patients refused surgery, due to significant symptomatic relief (two patients in CRT group, four patients in TNT group). They were eventually evaluated as cCR by MDT and received watch-and-wait strategy. Table 1 shows the patients characteristics. Age, sex, ECOG performance status, clinical stage, and distance from anal verge were similar between the two groups (all *p* > 0.05).

### 3.2. Neoadjuvant Administration and Toxicities

In CRT group, all the patients received oral capecitabine for 25 days. In TNT group, 111 (95.7%) received a full three-cycle course of CAPOX. There were five patients (4.3%) who received two cycles of CAPOX for the reason of grade 3 neutropenia. The mean number of CAPOX cycles was three cycles (SD 0.2). The most common grade 3 acute adverse events that were associated with the neoadjuvant administration were neutropenia in 18 patients (9.1%; six in CRT group and twelve in TNT group), thrombocytopenia in 13 patients (6.6%; four in CRT group and nine in TNT group), diarrhea in 11 patients (5.6%; three in CRT group and eight in TNT group), and rectal pain in 11 patients (5.6%; four in CRT group and seven in TNT group). Grade 3 anemia and radiation dermatitis were observed in nine patients (4.6%; three in CRT group and six in TNT group), respectively. Only one patient had grade 3 vomiting in TNT group. The proportion of patients of CRT group experiencing adverse events was lower than the TNT group during the neoadjuvant treatment. There was no statistically significant difference in the acute adverse events between the two groups. Table 2 summarizes the grade 3 acute toxicities of neoadjuvant treatment. No grade 4 or serious adverse events were observed. No deaths occurred during chemoradiation and consolidation chemotherapy.

### 3.3. Pathologic Response

Table 3 describes all pathologic findings. In the CRT group, 10 (12.8%) of 78 patients who underwent surgery achieved a pCR, whereas 36 (32.7%) of 110 patients had surgery and a pCR in the TNT group (*p* = 0.002). Patients of the CRT group and TNT group had similar ypTNM classification (stage Ⅰ 29.5% vs. 23.6%, *p* = 0.368; stage Ⅱ 30.8% vs. 28.2%, *p* = 0.701; stage Ⅲ 26.9% vs. 15.5, *p* = 0.054) and tumor regression grade (TRG) scale (TRG1 34.6% vs. 26.4, *p* = 0.223; TRG2 30.8% vs. 28.2%, *p* = 0.701; TRG3 21.8% vs. 12.7%, *p* = 0.099). There was no statistically significant difference in venous invasion (26.9% vs. 21.8%, *p* = 0.419) and perineural invasion (28.2% vs. 23.6%, *p* = 0.479) between the patients in the CRT group when compared with those in the TNT group.

### 3.4. Surgery and Surgical Morbidity

A total of 188 oncological rectal resections with TME were available for the complete pathologic examination and morbidity comparisons. Table 4 illustrates the surgical results from our study. In the two groups, 70.2% (132/188) and 23.4% (44/188) of patients underwent low anterior resection and abdominoperineal resection, while the other 12 (6.4%) patients underwent a Hartmann procedure. The rate of APR was higher in the TNT group (31.8 vs. 11.5%, *p* = 0.002). There was a trend that the mean operative time and blood loss was longer and greater in the TNT group (195.1 ± 11.3 min. vs. 180.5 ± 12.3 min., *p* = 0.132; 122.1 ± 20.2 mL vs. 102.1 ± 15.2 mL, *p* = 0.343), respectively. There was no statistically significant difference in pelvic infection, anastomotic leakage, bowel obstruction, wound infection, and pulmonary infection (all *p* > 0.05). Only two (1.8%) patients had anastomotic bleeding in the TNT group. The mean length of hospital stay was similar across the two groups (*p* = 0.869).

## 4. Discussion

The therapeutic approach of LARC primarily aims to improve local control and long-term overall survival. Recently, TNT has been attracting increasing interests [11,12,13,14,15,16]. In these studies, the authors discovered the following advantage: moving systemic therapy earlier than CRT in order to address possible microscopic metastatic disease, increased treatment compliance, assessed the sensitivity of chemotherapy in vivo, and avoided the discomfort of patents undergoing chemotherapy with a stoma. TNT includes different strategies, the most reasonable sequence of the induction chemotherapy, concurrent CRT, and consolidation chemotherapy is still controversial.

In our study, we showed that our 32.7% pCR rate in TNT was significantly higher than the 12.8% rate in CRT (*p* = 0.002). These results were comparable with those of previously published studies regarding induction chemotherapy [11,13,14]. In a recent study that was published by Cercek et al., [13], which examined 308 patients receiving TNT (introduction fluorouracil-and oxaliplatin-based chemotherapy model), the pCR rate was 35.7% in the TNT cohort compared with 21.3% in the CRT with planned adjuvant chemotherapy cohort. However, in a large phase Ⅲ Polish trail, patients with consolidation chemotherapy only had a 16% pCR rate [16]. In this trail, patients were randomly assigned to two treatment groups: one with short-course radiotherapy (5 × 5 Gy), followed by three cycles of chemotherapy with fluorouracil and oxaliplatin and the other with standard CRT. In fact, a recent systematic review of factors affecting tumor response to neoadjuvant therapy in over 4700 patients undergoing Phase II and Phase III trials has shown that a dose of >45 Gy was significantly associated with increased rates of complete tumor response [23]. In our study, all of the patients that underwent IMRT received a total dose of 50.0 Gy in the two groups, which may be one of the factors leading to a higher rate of pCR after neoadjuvant therapy in rectal cancer.

In my study, it was uncertain that the contribution of consolidation chemotherapy or the time from chemoradiation to surgery to pCR rate. It is well established that longer intervals from the completion of chemoradiotherapy to surgery are associated with an increase in pCR rate [24,25]. However, several recent retrospective and small-sample studies have found that consolidation chemotherapy could also improve pCR rate in the same time interval as CRT [26,27]. In these studies, the patients received two cycles of consolidation chemotherapy with capecitabine or CAPOX between CRT and surgery. The results showed that consolidation chemotherapy was significantly associated with pCR (*p* < 0.05) in univariate analysis. Garcia Aguilar et al., reported that consolidation chemotherapy during CRT for distal rectal cancer led to a sustained decrease in tumor metabolism when compared to standard CRT regimen [18]. In their study, the clinical assessment of tumor response in both groups was performed at 12 weeks after radiotherapy completion. In the present study, the reason for improvement of pCR rate by TNT might be the longer interval and addition of chemotherapy.

Some new data on TNT were presented at the 2020 American Society of Clinical Oncology annual meeting. In the RAPIDO trial, Geke Hospers et al., reported that a higher pCR rate (27.7% vs. 13.8%, *p* < 0.001) could be achieved with preoperative short-course radiotherapy, followed by chemotherapy and TME when compared to conventional chemoradiotherapy. They also found lower rates of disease-related treatment failure and distant metastases in the experimental arm at three years. In the OPRA trial, preliminary results showed that CRT, followed by consolidation chemotherapy, resulted in a numerically higher watch and wait strategy rate as compared to induction chemotherapy, followed by CRT. Both of the studies confirmed the efficacy of consolidated chemotherapy. The results of the RAPIDO trial did not indicate whether the improvement of pCR was due to the effect of time interval or consolidation chemotherapy. We speculated that it might be the result of multiple factors, including the effect of extended chemotherapy, continuous cellular lysis that is induced by radiation, and changes in tumor microenvironment after chemoradiotherapy. The OPRA trial has shown acceptable preliminary results, and it is worth exploring further.

The pathologic complete response following neoadjuvant chemoradiotherapy and interval proctectomy, in patients with rectal cancer, was associated with excellent long-term survival and low rates of local recurrence and distant disease [28]. Park et al., reported that the tumor response (complete v intermediate v poor) to neoadjuvant chemoradiotherapy among patients with locally advanced rectal cancer undergoing radical resection was associated with five-year RFS (recurrence-free survival) (90.5% vs. 78.7% vs. 58.5%; *p* < 0.001), five-year DM (distant metastasis) rates (7.0% vs. 10.1% vs. 26.5%; *p* < 0.001), and five-year LR (local recurrence) rates (0% vs. 1.4% vs. 4.4%; *p* = 0.002) [29].

In our present study, lengthening the neoadjuvant treatment time by delivering CAPOX before operation did not increase the risk of disease progression. The rates of distant metastasis diagnosed during neoadjuvant treatment were equivalent between the two groups. No grade 4 or serious adverse events were observed in our study. Although TNT group had higher grade 3 toxicity rate than CRT group, there was no statistically significant difference between them. Our results were consistent with previously published evidence [12,30]. In a phase 2 trial that was performed by Fernandez-Martos [12], the addition of induced chemotherapy did not increase the incidence of grade 3–4 adverse events in the neoadjuvant treatment. In another study [30], a similar conclusion was reached, where consolidation chemotherapy did not increase the incidence of complications after chemoradiotherapy.

Regarding the safety of surgery, our data showed that the mean operative time and blood loss were longer and more in the TNT group (195.1 ± 11.3 min. vs. 180.5 ± 12.3 min., *p* = 0.132; 122.1 ± 20.2 mL vs. 102.1 ± 15.2 mL, *p* = 0.343), which may be related to more APR patients in TNT group (31.6% vs. 13%, *p* < 0.05). We believe that the higher APR rate is due to more patients with low rectal cancer in the TNT group (tumor height from anal verge less than 5 cm: 74 (63.8%) vs. 41 (50.6%), *p* > 0.05). The other specific complications, such as pelvic infection, anastomotic leakage, bowel obstruction, wound infection, and pulmonary infection, did not differ between study groups. Garcia-Aguilar et al., conducted a multicenter study [31], and they found that delivering systemic chemotherapy after chemoradiation did not increase the risk of surgical complication.

The chemoradiation-to-surgery interval was prolonged in the TNT group. Although the optimal interval between completion of neoadjuvant chemoradiotherapy and surgery in rectal cancer is controversial [32,33], increasing evidences showed that operating 12 weeks after radiotherapy is safe with improved treatment response [34,35]. In our study, the interval between radiotherapy and surgery in TNT group was 12 weeks, which did not increase the surgical complications. Habr-Gama et al., found that patients undergoing surgery 12 weeks or more from CRT completion was safe and did not negatively affect survival [34]. The time from completing neoadjuvant therapy to surgery was almost at 12 weeks in Cercek et al., study [13].

We did not routinely evaluate cCR to neoadjuvant therapy in two groups. Six patients (two patients in CRT group, four patients in TNT group) refused operation because of cCR and they underwent observational management. In 2004, Habr-Gama et al., published that the OS and DFS at five years ended up being higher in the observation group than resection group [36]. OnCoRe et al., from the UK performed a prospectively study supporting watch-and-wait approach [37]. They demonstrated that there was no statistical difference between watch and wait and surgical resection in three-year DFS and OS. In addition, the colostomy-free survival was significantly better in the observational group (47% vs. 74%, *p* < 0.001). The evaluation of clinic complete response according to current adopted criteria has low sensitivity [38], so how to identify the patients with a cCR who may potentially benefit from the nonoperative management is still a problem.

Several limitations of our trail deserve mention. First, this study was a retrospective study, which might have selection bias. Although our findings lend support to TNT (consolidation chemotherapy mode), they should still be regarded as exploratory and in need of confirmation in a prospective randomized trial. Second, our study revealed that TNT strategy had a high pCR rate. Although pCR is associated with improved long-term outcomes [29,30], further investigation with mature follow-up and survival data are warranted. Third, patients with cCR were still recommended to receive radical surgery. The watch-and-wait approach to the management of cCR in rectal cancer is often the first concern of a patient, and we, as clinicians, are obliged to discuss these options with our patients.

## 5. Conclusions

This preliminary analysis shows patients undergoing CRT, followed by three cycles of CAPOX, is safe and tolerable. This consolidation chemotherapy model is associated with high rates of pathological complete response. The long-term follow-up is necessary to be evaluated in a future prospective randomized trial for essential information on the optimization of the TNT model.

## Figures and Tables

**Table 1:** Patients’ Characteristics.

Characteristics	Patients, No. (%)		
	CRT (n = 81)	TNT (n = 116)	*p* Value
Age(years)	60.2 ± 11.4	59.7 ± 11.8	0.859
Sex			
Male	47 (58.0)	73 (62.9)	0.487
Female	34 (42.0)	43 (37.1)	
Tumor height from anal verge (cm)			
<5	41 (50.6)	74 (63.8)	0.181
5–10	31(38.3)	33 (28.4)	
>10	9 (11.1)	9 (7.8)	
ECOG			
0	71 (87.7)	106 (91.4)	0.394
1	10 (12.3)	10 (8.6)	
cT stage			
T2	4 (4.9)	9 (7.8)	0.721
T3	64 (79.0)	84 (72.4)	
T4a	10 (12.3)	19 (16.4)	
T4b	3 (3.8)	4 (3.4)	
cN stage			
cN0	23 (28.4)	24 (20.7)	0.212
cN positive	58 (71.6)	92 (79.3)	
Clinical stage			
Ⅱ	15 (18.5)	28 (24.1)	0.347
Ⅲ	66 (81.5)	88 (75.9)	
metastasis	1 (1.2)	2 (1.7)	1.000

ECOG, Eastern Collaborative Oncology Group.

**Table 2:** Sever Acute Toxicity of Neoadjuvant Treatment.

Grade 3 Acute Toxicities of Neoadjuvant Treatment	CRT (n = 81)	TNT (n = 116)	*p* Value
Neutropenia	6 (7.4)	12 (10.3)	0.481
Anemia	3 (3.7)	6 (5.2)	0.889
Thrombocytopenia	4 (4.9)	9 (7.8)	0.433
Diarrhea	3 (3.7)	8 (6.7)	0.519
Vomiting	0	1 (0.9)	
Radiation dermatitis	3 (3.7)	6 (5.2)	0.889
Rectal pain	4 (4.9)	7 (6.0)	0.989

**Table 3:** Pathologic Examination of Resected Specimens.

Tumor Characteristic	CRT (n = 78)	TNT (n = 110)	*p* Value
pathological complete response	10 (12.8)	36 (32.7)	0.002
ypTNM classification			
Ⅰ	23 (29.5)	26 (23.6)	0.368
Ⅱ	24 (30.8)	31 (28.2)	0.701
Ⅲ	21 (26.9)	17 (15.5)	0.054
tumor circumferential margin < 1 mm	0	1 (0.09)	
TRG scale			
0	10 (12.8)	36 (32.7)	0.002
1	27 (34.6)	29 (26.4)	0.223
2	24 (30.8)	31 (28.2)	0.701
3	17 (21.8)	14 (12.7)	0.099
Venous invasion	21 (26.9)	24 (21.8)	0.419
perineural invasion	22 (28.2)	26 (23.6)	0.479

TRG, tumor regression grade.

**Table 4:** Surgical Results.

Items	CRT (n = 78)	TNT (n = 110)	*p* Value
Type of surgery			
LAR	60 (77.0)	72 (65.5)	0.002
APR	9 (11.5)	35 (31.8)	
Hartmann	9 (11.5)	3 (2.7)	
Pelvic infection	2 (2.6)	6 (5.5)	0.548
Anastomotic leakage	4 (5.1)	8 (7.3)	0.772
Anastomotic bleeding	0	2 (1.8)	
Bowel obstruction	2 (2.6)	5 (4.5)	0.752
Wound infection	3 (3.8)	6 (5.5)	0.871
Pulmonary infection	1 (1.3)	3 (2.7)	0.87
Operation time(minutes)	180.5 ± 12.3	195.1 ± 11.3	0.132
Blood loss(milliliters)	102.1 ± 15.2	122.1 ± 20.2	0.343
Hospital stay(days)	7.8 ± 3.2	6.9 ± 3.8	0.869

LAR, low anterior resection. APR, abdominoperineal resection.
